# “I Don’t Hate All Women, Just Those Stuck-Up Bitches”: How Incels and Mainstream Pornography Speak the Same *Extreme* Language of Misogyny

**DOI:** 10.1177/1077801221996453

**Published:** 2021-03-22

**Authors:** Alessia Tranchese, Lisa Sugiura

**Affiliations:** 1 University of Portsmouth, UK

**Keywords:** Incels, pornography, online misogyny, Corpus Linguistics

## Abstract

This article seeks to establish the connection—via shared discourse—between Incels and mainstream pornography. With an interdisciplinary approach which involves a Corpus Linguistics analysis of Reddit forum data, research into digital behaviors, and a feminist critique, this article focuses on the commonalities between the language of pornography and that of Incels. In doing so, it demonstrates how both pornography and Incels are different manifestations of the same misogyny. The findings of this study highlight the normalization of violence against women (VAW), which continues to be endemic in society, enabled and exacerbated by contemporary technologies.

## Introduction

This article seeks to establish the connection—via shared discourse—between those who identify as involuntary celibates online (henceforth Incels) and mainstream pornography. Using an interdisciplinary approach involving linguistic analysis of Reddit forum data—more precisely the r/incels Subreddit—informed by research into digital behaviors and feminist analysis, we demonstrate how both mainstream pornography and Incels are different manifestations of the same deep-rooted misogyny, enabled and exacerbated by contemporary technologies. It is not the aim of this article to establish a causal link between the watching of pornography and negative behavioral change, nor is it our intention to cast judgments on people’s sexual practices. We want to move beyond a simplistic pro-porn or anti-porn dichotomy that discourages debate, and instead provide *evidence* of its commonalities with other practices that are more often seen as aspects of the ongoing normalization of violence against women (VAW) and whose misogynistic nature, therefore, tends to be taken more seriously. We start by comparing the language used by Incels on the news aggregator site Reddit with the language of a larger sample of Reddit threads. The linguistic analysis is carried out using a bottom-up mixed-method approach which does not impose predetermined categories onto our data but, instead, allows themes and patterns to arise gradually from our data (Baker et al., 2013). Thus, a quantitative analysis using Corpus Linguistic is carried out to process a large amount of linguistic data and observe dominant patterns and themes based on word frequency and word co-occurrence incidence. This is followed by a qualitative investigation which involves the contextualization of linguistic data in the broader context of Incel ideology. In this phase, the findings from the linguistic analysis are also evaluated against what the existing literature has argued about mainstream pornography. This cross-evaluation highlights how both Incels and mainstream pornography share discourses that reflect a broader societal misogyny in which sex is punitive and strictly connected with women’s submission.

Incels have recently generated large amounts of interest, both inside and outside academia (Ging, 2017a; Jaki et al., 2018; Marwick & Caplan, 2018; Nagle, 2017) due to concerns about their incitement of hatred and violence, particularly against women. Some research has also established links between these men and pornography. For example, Burnett (2019) has shown that #nofap (abstention from masturbation, particularly to pornography) is a common theme among these men, arguably pointing to an underlying concern about pornography addiction. Jaki et al. (2018) have suggested an even stronger link between the two, by stating that the discussions about pornography on Incel forums show their misogynistic stance. However, debates about Incels and mainstream pornography mostly run parallel to each other.

On one hand, Incels are seen as “one of the internet’s most dangerous subcultures” (Beauchamp, 2019) and an expression of “extreme feelings of misogyny and hatred” (The Fifth Estate, 2019). On the other, pornography is widely perceived as non-threatening and harmless because it is seen as fiction (MacKinnon, 1984), and, arguably, its influence is often underestimated. Yet, the effects that this large exposure, at increasingly younger ages, is having on society is concerning. For instance, for a large number of children and young people, including girls and young women, pornography now plays a key role in their sexual education (Lim et al., 2017). Some of the most popular categories accessed through pornographic websites include violence and aggression toward (young) women (Bridges et al., 2010), and research has shown that frequent exposure to pornography increases the likelihood of engagement in risky sexual behaviors, self-objectification, and endorsement of rape myths (Flood, 2009; Tylka & Kroon Van Diest, 2015). Despite increasing evidence that mainstream pornography is misogynistic and harms women (MacKinnon, 1984; Tyler, 2011) and that its content is becoming more and more extreme, radical feminist arguments against it are often labeled as “sex-negative” (Jensen, 2016, p. 4), while pornographic materials remain widely available and easily accessible, particularly on the web (Ezzell et al., 2020). As a matter of fact, at the time of writing, pornographic websites such as PornHub.com feature among the top 30 websites in the United Kingdom and is more popular than the U.K. government website (“Top Sites in UK,” 2020). Arguably, it is this very widespread availability and ease of access that has contributed to the establishment of pornography as a mainstream, widely accepted, and normalized form of “harmless fiction.”

However, separating “extreme” versions of an ideology from more “mundane” ones creates an artificially clear distinction between the “crazy fringes” and mainstream ideologies that, instead, are based on the same assumptions. The extreme/non-extreme dichotomy is misleading, as it obscures systems of oppression and “ordinary” misogyny—particularly online, where such boundaries can be harder to demarcate—that have become socially sanctioned and normalized, like mainstream pornography. Moreover, this arbitrary division absolves the “non-misogynistic” majority which is allowed to express reprimand for and rejection of “extreme” misogyny, while presenting itself as morally irreprehensible (Ferber, 1996). Therefore, in this article, both mainstream pornography and Incels are conceptualized as expressions of the same misogynistic ideology and, more precisely, as forms of VAW and, to expose the connection between “extreme” and “mundane” practices, we use language as entry point into the conceptual and ideological contexts that underpin *both*.

Sexist language cannot be said to have been created by modern, mainstream pornography or by Incels; in fact, this is a semantic set that, as suggested by Penelope (1990), “seems to be able to be expanded infinitely” with new visual and verbal manifestations (p. 121). However, pornography has drawn on and added to it, and, thanks to the ubiquity of the internet, this imagery has spread beyond its sphere, leading to what Dines (2010) and Shor (2018) define as an escalating “pornification” of society. The accessibility, affordability, and anonymity (Cooper, 1998) of pornography viewed through the internet—particularly “gonzo” pornography “which depicts hard-core, body-punishing sex in which women are demeaned and debased” (Dines, 2010, p. xi)—have exacerbated the normalization of the dehumanization of women. Similarly, Incels have drawn on and added to the repertoire of sexist language available to them. The development of this community was also favored by the internet, which has allowed its members to form their own shared values, meanings, and ideologies that appear contrary to wider society (King, 2008). However, the findings of our study demonstrate the shared discourse and commonalities between the Incel “fringe” and the overwhelming “ordinary” majority that preserves gender inequality through normalized practices such as mainstream pornography. We suggest that both should be understood as elements on the continuum of VAW (Kelly, 1988; McGlynn et al., 2017). While differences between mainstream pornography and Incels exist—the former is a lucrative industry and, technically, a form of filmmaking, whereas the latter are an online community of men who articulate their frustrations in shared forums—they speak a common language which highlights the structural misogyny that underpins both. It is perhaps unattainable to establish with absolute certainty how these similarities have developed– that is, if Incels have learned about sex and women through pornography or if pornography is their weapon against women. Yet, they reveal how both practices contribute to expanding and reinforcing each other’s discourses and range of misogynistic practices. To expose the commonalities between VAW, pornography, and Incel misogyny, we chose not to censor the offensive language used in these spaces (Jane, 2014, p. 536).

## Online Misogyny and VAW

Incels are often considered a misogynistic “fringe” because of their explicit sexism and hatred for women (Tait, 2018). Their misogyny is often seen as an issue of a small group of deviant individuals, whose problematic attitudes toward women are exclusively attributed to their personality, as individual deviance or mental illness, and without connection to structural misogyny or patriarchal systems of socialization (Manne, 2018). In this sense, Incels are not conceptualized differently from rapists: deviant men who engage in “extreme” acts of misogyny because of their individual pathology (Malamuth, 1981). Drawing on Citron and Norton (2011), who framed online abuse as a restriction of women’s civil engagement and abuse of their “digital citizenship,” Lewis et al. (2017) have highlighted further similarities between offline violence and online abuse against women, such as the fact that both have the effect of instilling fear into women, and silencing them, especially through the threat of sexualized violence, but also through exclusion, disdain, or discrediting. This policing of women’s behavior functions as a reminder of who is in control and who dictates the boundaries within which women are free to move (Megarry, 2014), while men’s freedom remains unrestricted. In the case of offline violence, these boundaries constitute quite literally a demarcation of men’s territory and a limitation of women’s freedom of movement (Vera-Gray, 2018).

On r/incels, women are often excluded from conversations. As a consequence, the amount of direct abuse displayed toward them on the site is relatively low; nevertheless, the level of misogyny is high. Therefore, these public discussions are a valuable tool to investigate the motivations, reasoning, and conceptual frames that support online misogyny in the Incel community—rather than to study direct harassment—and to highlight the commonalities they share with the belief systems that sustain other forms of (online and offline) VAW, including mainstream pornography. In fact, we argue that, like offline violence, online abuse also happens on a continuum; this spectrum, however, does not simply range from unpleasant, sporadic, nonthreatening (direct or indirect) messages to frequent, highly threatening, hateful content (Lewis et al., 2017, p. 1469). On the contrary, the continuum also involves misogynistic discourse and practices considered noncriminal or nonthreatening because they are seen as “fiction,” as satire/cultural in-jokes (Zillmann, 1983), or because they do not entail a direct interaction between users. Breaking the continuum to single out “exceptional behavior” protects more mundane forms of misogyny from scrutiny. Thus, both mainstream pornography and Incel misogyny should be incorporated in wider forms of VAW.

## Pornography and VAW

The link between mainstream heterosexual pornography (the most popular and widespread type of pornography and the one that is being discussed here) and (online) VAW is not necessarily an obvious one. In fact, the conceptualization of pornography as generally and inherently misogynistic is not without criticism (Strossen, 1996). Admittedly, our claim is that it is the way in which male–female relationships (including male and female sexuality) are understood in society that is misogynistic and is reflected in a series of practices shaped by this structural misogyny. Several studies (Brownmiller, 1975; Dines, 2010; Dworkin, 1981; Paul, 2005) have highlighted that what makes modern pornography, particularly online mainstream (gonzo) pornography, misogynistic is its tendency to objectify and dehumanize women. Dworkin and MacKinnon (1988) famously defined pornography as “the sexually explicit subordination of women, graphically depicted, whether in pictures or in words” (p. 101). The verbal and visual subordination of women takes several forms. For example, Bridges et al. (2010) found that a large number of popular online pornographic videos contain images of physical aggression toward women, including spanking, gagging, and slapping. These appeared particularly in scenes of degrading sexual practices, such as ass-to-mouth (ATM) penetration, in which a penis goes from a woman’s anus to her mouth without washing. They also identified a correlation between the physical and verbal aggression of women, with the latter representing a strong predictor of the presence of the former. While the verbal sexual degradation of women was not invented by pornography, but is a well-documented phenomenon (Penelope, 1990; Romaine, 1999), pornography draws heavily on this language, with name-calling being one of the most common forms of verbal aggression (Bridges et al., 2010). However, although *cunt, bitch, whore, slut* continue to be some of the most commonly used forms of language abuse against women in pornographic videos (Bridges et al., 2010; Dines, 2010; Shor, 2018), pornography also contributes to the expansion of this repertoire beyond the domain of pornography, with the popularization—through, for example, websites such as Urban Dictionary (Ging, 2017b; Ging et al., 2019)—of new terms, and associated images, that degrade and objectify women (e.g., *cumdumpster, fuckhole, meathole, fucktubes, cum swapping, money shot, bukkake*). Pornography, therefore, is both a perpetuating and innovative practice of misogyny.

Reducing women to objects in pornography is a precondition to make the violence they endure look acceptable. When women stop being people, acts of violence against them stop being harmful, as objects cannot be harmed. The legitimation of VAW in pornography is also supported by two latent assumptions. First is the idea that pornography is a “distortion, reflection, projection, expression, fantasy, representation or symbol” (MacKinnon, 1984, p. 326) of reality and, therefore, not *real*. Yet, “fantasy expresses ideology” (MacKinnon, 1984, p. 327); it expresses the reality of the subordination of women entrenched in the way we understand sex, in and out of pornography. In fact, the second assumption that legitimizes VAW in pornography– that is, that “women enjoy sexual mistreatment” (Dines, 2010, p. 64), consent to their own humiliation, and never say “no” to degrading acts– shows how eroticization and women’s subordination are strictly connected, with the latter becoming “socially real” through its enactment in pornography (MacKinnon, 1984, p. 327). The illusion of consent covers the sexist nature of these acts and allows the refusal of sex not only to become indistinguishable from the desire of sex, but also normalized and eroticized. In pornography, even women’s “no” is part of the fantasy, and force is no longer seen as force “because it is inflicted on women and called sex” (MacKinnon, 1984, pp. 340–343). Under this pretense, almost everything becomes justifiable, including degrading and violent acts such as shoving a woman’s head down the toilet, gagging her, or making her ingest her own vomit. While women become powerless in pornography—as willing actors who ask to be acted upon—men become powerful and always obtain as much sex as they want, how they want it. For a short time, men “get to see what life would look like if only women unquestionably consented to men’s sexual demands” (Dines, 2010, p. 63).

## Pornography and Online Misogyny as Speech Acts

Pornographic acts also have tangible effects on real people (Clark-Flory, 2018). As suggested by Lewis et al. (2017), “the ‘real’ and the virtual are not separate experiential realms; activities that take place in the virtual world are still experienced as reality, with material consequences” (p. 1465). In this sense, pornography can be understood as a speech act with both an illocutionary and perlocutionary dimension (Austin, 1962; Langton, 1993, 2012). Through its illocutionary force pornography can *subordinate* women, *endorse* their degradation, *legitimate* misogynistic attitudes by depicting their dehumanization as sexual objects, by showing them enjoying pain, humiliation, and violence, and by portraying them “in postures of sexual submission or servility or display; reduced to body parts [. . . ] in a context which makes these conditions sexual” (Dworkin & MacKinnon, 1988, p. 36). However, it is through its perlocutionary effect—accorded by its (financial) power and authority—that pornography can also *cause* and/or *perpetuate* subordination, by generating changes in feelings, thoughts, attitudes, or behaviors. For example, pornography can perpetuate hatred toward women, and it can influence one’s interpretation of women’s subalternity as natural and inevitable (Langton, 1993); it can also have the effect of silencing women through the systematic suppression of their voices, sexuality, and humanity. Thus, as a speech act, pornography performs and reifies gender inequality and, due to its pervasiveness, its ramifications spread beyond those who directly watch it. Langton (2012) further suggests that pornography shares these features with (misogynistic) hate speech, in that both can *depict, cause*, and *be* subordination at the same time. Thus, we argue that, like pornography, Incel misogyny is a speech act whose perlocutionary effect is to silence women, police their behavior and, ultimately, harm them.

## Digital Technology, Online Communities, and Misogyny

Digital technologies are embedded into our everyday lives due to the symbiotic relationship between technology and society (Powell et al., 2018). While, arguably, digital technologies (or the internet) and, consequently, many of the practices that take place online are shaped by structural misogyny (technology has been created by people who have been conditioned by patriarchal structures), these are not causal in the abuse of women (they do not pre-exist hatred against women). Nevertheless, digital technologies have created new ways for hatred to advance and disseminate in unprecedented ways. In particular, online communities and virtual platforms have provided the means for collective animosity to advance on an exponential scale. The structure of the internet, with its searching capabilities, algorithmic politics (Ging, 2017a; Massanari, 2017)—which prioritize the interest of straight, White males—and other affordances such as anonymity and a myriad of topics and communities, have made it easy to find others with similar interests and/or belief systems. As Perry (2001) highlights, the internet is a means of connecting like-minded individuals. For example, members of hate movements that were previously fractured were brought together and facilitated in the process of creation of a collective identity and empowering sense of community. The connectivity and social components of the internet have also played a significant role in the cross-pollination of misogyny across platforms, enabling a “networked misogyny” (Banet-Weiser & Miltner, 2016) to flourish, connecting different groups linked by the same ideologies regarding women.

## Incels

The term Incel stands for “involuntary celibate” and was created in 1993 by a female student—Alana—who, on her website, described Incel as “anybody of any gender who was lonely, had never had sex or who hadn’t had a relationship in a long time” (Taylor, 2018). However, the term has now been appropriated by a group of aggrieved men to exclude women and propagate misogyny. Incels have a strict social hierarchy, mostly based on appearance and wealth. With Incels at the bottom, this hierarchy includes Chads and Stacies. Chads are men who are “high status,” rich, muscular, and have sex with as many women as they want. Chads are the embodiment of the alpha male, and diametrically opposed to Incels. Stacies are attractive women who will only have sex with Chads (Incel Wiki, 2019).

Incels are part of a larger backlash against feminism propelled by what has been defined as the “manosphere” (Marwick & Caplan, 2018), that is, groups of men whose ideology is informed by misogynistic tropes aimed at silencing feminist voices (Faludi, 1991; Venäläinen, 2019). These tropes include the idea that feminism has corrupted society and is unnecessary because equality has been achieved, and that women’s equality is detrimental to men and, therefore, men need to retaliate against this misandrist culture to preserve their very survival. Blaming feminists for their misery gives these men license to abuse in order to redress the balance (Jhaver et al., 2018). Although the main interests of each group within the manosphere may differ, their common language creates a unified identity. While Incels are not an isolated phenomenon, they are considered particularly dangerous as they were associated with a series of killings committed in Toronto and Edmonton, Canada, and in the United States, in Oregon, Florida, New Mexico and, perhaps most notoriously, in California. The latter were killings committed in the community of Isla Vista by Elliot Rodger, an Incel apparently motived by his “extreme” misogyny. In a video (Kron 4, 2014) posted by Rodger just before the attack, he explained his motives:

I’m 22 years old and I’m still a virgin. [. . .] College is the time where everyone experiences those things such as sex and fun and pleasure. [. . .] I don’t know why you girls aren’t attracted to me but I will punish you all for it. [. . .] On the day of retribution, I am going to enter the hottest sorority house of UCSB and I will slaughter every single spoiled stuck-up blonde slut I see inside there. [. . .] You will finally see that I am in truth the superior one, the true alpha male.

The data used in this analysis were collected from an Incel forum—r/incels, once reportedly the most active Incel community online (Hathaway, 2017)—on Reddit. Reddit is a U.S. social news aggregation and discussion forum. Members post content to the site, which is then voted up or down by other members. Posts are organized by subject into user-created boards called Subreddits. The r/incels Subreddit (which had roughly 40,000 members—Hauser, 2017) was removed by moderators in October 2017, when Reddit introduced a new policy to ban “content that encourages, glorifies, incites or calls for violence or harm against an individual or a group of people” (Hauser, 2017). Some of the activity that led to the Subreddit being taken down included advice on how to get away with rape.

## Methodology: Corpus Building and Analysis

This study is based on a comparison between two datasets: a study corpus (henceforth IncelCorpus) and a reference corpus (henceforth RedditCorpus). The IncelCorpus was collected from Pushshift.io (http://files.pushshift.io/), an open data initiative that provides access to social media companies such as Twitter and Reddit. The IncelCorpus contains 13,783,206 words and includes posts published on r/incels between June 2016 and March 2017. The RedditCorpus was collected extracting posts and comments from Reddit using the Reddit API. The timeframe in this corpus varies in each Subreddit, since with the Reddit API only a maximum of 10 pages per Subreddit can be retrieved; therefore, time scales depend on how quickly (or slowly) users post new threads to the Subreddit. This corpus contains a collection of 688 Subreddits on disparate topics, amounting to 89,386 posts, and ca. 55 million words.

The RedditCorpus was used as a normative corpus (Rayson, 2003) that represented the standard of how users talk on Reddit. The advantage of using a specific RedditCorpus as reference corpus is that, in the comparison, the latter is more likely to highlight features of language and discourse specific to Incels rather than to Reddit more generally (e.g., it is more likely to exclude words such as *Subreddit* or *moderator* than generic—but still prominent—terms in r/incels, like *women* or *people*). The analysis was conducted using the corpus analysis tool SketchEngine (http://sketchengine.co.uk/).

The comparison between the RedditCorpus and the IncelCorpus generated a group of keywords, that is, words that are “statistically significantly more frequent” (Baker et al., 2013, p. 27) in one corpus than in another. The measure employed to determine the statistical significance of the difference in frequency of occurrence of certain terms is Average Reduced Frequency (ARF; Savický & Hlaváčová, 2002). This method allows one to account for dispersion, discarding words that are very frequent only in some parts of the corpus in favor of words that are more homogeneously distributed throughout. After extracting the keywords, these were clustered together into semantic groups to highlight dominant topics. Due to space limitations, only the top 20 keywords are shown in the analysis, and a sample of words per semantic group is analyzed in detail using collocation and concordance analysis, which allowed us to conduct a closer reading and contextualization of texts. A “collocation” is the occurrence of two or more words within a short space of each other in a text. The measure of proximity used here is a maximum of four words intervening on either side of a search term (Sinclair, 1991, p. 170) and the measures of significance used were SketchEngine’s default settings for WordSketches which combine strength and frequency of co-occurrence. A WordSketch is a tool that provides a “summary of the word’s grammatical and collocational behaviour” (SketchEngine, n.d.). To retrieve a manageable amount of collocates, only words with a significance score >10 were considered. In addition to collocates, concordance lines were also extracted from the corpus. Concordance lines are instances of a word or phrase in context (Hunston, 2002) that show the use of that specific word or phrase in-text. Due to the large number of occurrences, concordance lines were reduced using SketchEngine’s random sampling function. All the examples are reported as published, without changing spelling or grammar.

This research took into account the ethical implications of using online user-generated content. Considering that the forum thread was no longer available in the public domain, accessing the forum data without attempting to contact the community on Reddit was the most ethical course of action, as the covert approach enabled research to be undertaken without risk or harm to the community (Sugiura et al., 2016). No personal data, including names, locations, or physical attributes were documented and any traceable information were anonymized or struck from the analysis.

## Analysis and Findings—Keywords and Salient Topics

Keywords offer an indication of the main topics discussed on r/incels, compared to Reddit in general. As shown in Table 1, the focus of Incels appears to be on four main semantic categories: groups of people, relationships, physical attributes, and verbs.

**Table 1.** Top 20 Keywords in IncelCorpus (the Font Denotes Lemmata, i.e., Canonical Word Forms That Include, for Example, Both Singular and Plural Forms).

People	Relationships	Physical attributes	Verbs
WOMAN, INCEL, GUY, MAN, GIRL, NORMIE, FEMALE, PEOPLE, MALE, CHAD	FUCK, SEX, DATE, RELATIONSHIP	UGLY, ATTRACTIVE	THINK, WANT, HATE
	Swearing: SHIT		

For reasons of space, here the focus will be mostly on the “people” and “relationship” groups. The terms in the latter group suggest a strong emphasis on both the sexual and dating sphere, while those in the former reflect the social hierarchy of Incels, with CHAD, INCEL, but also NORMIE, that is, ordinary people who do not belong to any of the other groups. Because of their uniqueness, terms such as CHAD and INCEL would be expected to be more salient than a generic term like WOMAN.However, WOMAN appears more frequently and widely than INCEL (with an almost double ARF score—13.5 vs. 7.9), showing that women are a focal point of discussion on r/incels. The absence of a sub-categorization of women equivalent to that of men (cf. absence of *Stacy* from the list, but presence of *Chad*), together with the frequent use of the plural *women*, suggests that women tend to be discussed as a homogeneous group, without much distinction.

## Analysis and Findings—Collocation Analysis

The following sections present a more granular analysis of WOMAN, CHAD, and FUCK in context, with a focus on their collocates, to evidence significant discursive patterns.

### Women

Table 2 contains the most frequent grammatical collocates of WOMAN.

**Table 2.** WordSketch for WOMAN (Examples in Brackets Show the Most Frequent Clusters for Each Collocate).

Modifiers of WOMAN	Verbs with WOMAN as subject	WOMAN is	Verbs with WOMAN as object
most (most women)^3^	be (women are)	whore (women are whores)	attract (attract women)
obese (obese women)	do (women don’t)	people (women are people)	hate (hate women)
many (many women)	want (women who want)	slut (women are sluts)	force (women)

*Most* and *many* are quantifiers that aggregate women together (van Leeuwen, 2008) and, like the plural form, reinforce the perception of women as a homogeneous group. Discussing women as a category not only denies their individual experiences and, arguably, facilitates their becoming objects, devoid of humanity and targets of hate, but also facilitates the creation and acceptance of clichés about their (supposed good or bad) “nature.”

Many users on r/incels discuss what *many/most women want, desire, like*. They do so in a way that imposes their male gaze (Mulvey, 1975) onto the category “woman,” because, while women’s presumed desires are eviscerated at length, women themselves are consistently kept out of the discussion, as their comments are often silenced through disbelief, verbal abuse, and/or requests to leave the forum (*Before women swarm me crying their argument “you’re wrong, big dicks hurt me, sex with them is painful, i like smaller ones and im happy with my partner.” No, please shut up and stop lying to men*). Due to the public, and, therefore, visible nature of the forum, these posts can also serve as a gatekeeping practice for anyone intending to challenge Incels’ ideology.

Another frequent modifier of *women* is *obese* (Table 2), particularly in the discussion of the success of obese women (as opposed to Incels) in finding (sexual) partners. This view is often supported by pseudo-scientific facts and dubious external data, suggesting that women, unlike men, are in high (sexual) demand and, therefore, even women who appear low on the “attractiveness scale” will find a (sexual) partner, while men who are not at the top of this scale are condemned to a life of celibacy. This is expressed clearly by one of the users on the forum: *There are many males that have literally zero options. [There is strong demand for even the ugliest of women.] (link to sluthate.com). The top 20% of men get the top 80% of the women, and the bottom 80% of men fight like dogs over the remaining 20% of women. This is why in a sexually free society, even the ugliest most deformed obese women can get males of average attractiveness*. In this system, women are the gatekeepers of sex, while Incels are their victims. As shown in the following, it is particularly feminism—with the sexual liberation of women—that Incels blame for their misery. Incidentally, this post is a bot that contains a link to a website affiliated with the manosphere (sluthate.com). While the discussion of bots is beyond the scope of this article, it is worth considering the role of automatic messages triggered by specific words in reinforcing online misogyny (Woolley & Howard, 2016).

WOMAN co-occurs often as grammatical subject of BE, DO, and WANT. As shown in Table 2, more than half the occurrences of *do* are, in fact, negations (*don’t*). The most common activities that women are presented as “not doing” (calculated using SketchEngine frequency option) are two mental processes: LIKE and WANT (Halliday, 2004). This means that women are often talked about in terms of what happens in their minds, rather than in terms of their actions in the external world. Thus, despite their factual and pseudo-scientific tone, these discussions are speculative, particularly when they occur alongside aggregation (e.g., *Because the majority of modern women don’t want a loving boyfriend*). These conjectures often revolve around the type of men that women are supposedly attracted to (e.g., *Women don’t like ugly men who aren’t rich, because they aren’t good providers*), or around women’s sexuality, including the suggestion that women enjoy abusive relationships and aggressive sex.

Like in pornography, where the tendency is for female performers not to say “no,” but to show pleasure when subjected to physical aggression, Incels believe that aggression and female pleasure are connected (e.g., *Most women don’t like it soft: they like it rough, hard, and deep*). In some cases, the strong presence of negation, combined with the collectivization of women, signals resistant discourse (e.g., *Women don’t like being raped BTW*). Yet, the very need for this resistance is indicative of underlying presuppositions among Incels about women and what they “really” want. Furthermore, when Incels discuss what women *like* and *want*, they indicate that it is specifically *Western* *modern women* *(Western is the most common attribute of women want)*—in other words, those they associate with feminism—who enjoy “deviant” practices, as shown by the most frequent clusters with *women want: women only want* or *women only truly want* (e.g., *Modern Western women only want the worst men; Women want only the best looking men, and thanks to feminism they can have them)*.

Scholars (Jensen, 2011; Makin & Morczek, 2015) have suggested that mainstream pornography—with its unceasing exploitation of women—encourages the acceptance and propagation of “rape culture,” that is, attitudes, behaviors, and norms connected with VAW. By raising tolerance levels for aggressive sex, it blurs the line between violence and sex, and crucially, it suggests that women enjoy both. According to Jensen (2011), pornography teaches that “all women always want sex from men, women like all sexual acts that men perform or demand, and any woman who does not at first realize this, can be persuaded by force” (p. 30). Some comments on r/incels show that this is not just a prerogative of pornography: *But the deal is that they start liking it in the middle of the act or follow the guy around later like puppies on a leash. So rape is what modern Western women want.*

Table 2 also shows the most frequent attributes for women: *whores, people*, and *sluts*. The specifically sexually derogatory nature of this language is worth noting, as r/incels (like pornography) appears to be a site of constant (re)production of ways to linguistically degrade and objectify women. For example, Incels refer to women as *cumdump, cumrag*s, or *twatrags*—a term that indicates an old garment used to clean up bodily fluids following ejaculation. Another way in which Incels use sex to linguistically dehumanize women is through verbs that are normally applied to things such as soil or roads (e.g., *a 30yo woman who has been plowed by 150 different cocks*). The verb *plow* here, in addition to normally being applied to inanimate objects, also suggests violence (e.g., fields are plowed with sharp objects). The implication is not only that women are objects to be treated aggressively but also that the penis is a powerful weapon to be used against them. This discussion of women as objects used for sex or as receptacles of men’s penises and semen (*dumpsters, holes, tubes*) recalls some of the most popular images of mainstream pornography, like, for example, “bukkake,” in which any number of men ejaculate, often at the same time, onto a woman’s body, face, hair, eyes, ears, or mouth, or the “money shot” (Dines, 2010; Ging et al., 2019). Arguably, with men’s ejaculation, women do not just receive their semen; they also become receptacles of their rage and hatred.

Incels also dehumanize women by referring to them as objects, female humanoid organisms (or femoids) or animals, as shown by metaphors such as *women are fucking animals, women are shallow creatures, women are toxic wild beasts*. Perhaps the strongest indicator of the propensity of Incels not to see women as human beings is the frequent reminder on r/incels that *women are people* (e.g., *women are people literally just like you*). Arguably, the very need to state the obvious, that is, that women *are* people, is an intertextual reference to the common presupposition among Incels that women are something else (animals, objects, holes, containers). The dehumanization of women (for sexual pleasure) is part and parcel of “rape culture” (Jensen, 2011) and a major feature of mainstream pornography.

Further insight into how women are dehumanized on r/incels is provided by the analysis of verbs that have WOMEN as grammatical object. For instance, ATTRACT occurs most frequently in discussions about ways in which *men can attract women*. In the Corpus of Contemporary American English—https://www.english-corpora.org/coca/—ATTRACT is normally used in the context of entities that can bring financial advantage (e.g., *customers, investment*), or to refer to how (male) animals pull their (female) mates toward themselves (e.g., *the extraordinary plumages of the male suffice to attract several females*), or to talk about what one is attracted to (e.g., *I have always been sexually attracted to foreigners*). However, Incels do not use ATTRACT to discuss what they are attracted to, but to talk about what they can attract to themselves (i.e., women), in a way that resembles the literal meaning of the verb (i.e., the property of magnets to attract metal objects). Incels position both themselves and women as objects, but while they are the active force in this relationship, women are the passive element that—in spite of any potential resistance—gets pulled toward Incels, as shown in this comment: *You’ll find a lot more on how to attract women and improve self-esteem here /r/seduction*. This reinforces the impression that what Incels are really concerned with is not women’s desires, but their own self-esteem; for them, attracting is a utilitarian practice in which women are vehicles to boost their ego.

This sense of entitlement to and understanding of women’s bodies as objects (that must be obtained) also appears in the ways in which FORCE is used. While many users do not explicitly encourage forcing women to have sex or be in relationships with them, the discussion of the obvious suggests, again, that this is indeed a possibility for Incels. In fact, not forcing women is often linked to their *inability*, rather than their *unwillingness*, to do so (e.g., *I cannot force a woman to desire me or have sex with me so essentially I am screwed*). Women are once again positioned as gatekeepers of sex and, consequently, of Incels’ happiness. Incels, in contrast, are the victims: constantly starved for sex and fighting other men to gain access to women’s bodies that are scarcely available (to Incels).

Establishing their victimhood (Ging, 2017a) is key to transform “(not) *forcing* women” from a matter of women’s rights to a matter of Incels’ rights, as articulated in this post: *Arguably the fact that I do not have the freedom to force a woman to have sex with me is subjugation too*. To address this “injustice,” Incels suggest solutions that, while exploitative for women, they consider fair for themselves (e.g., legalizing prostitution*—It’s not like I want to force women to become prostitutes, they would all be voluntary*; limiting women’s [sexual] freedom—*The only way anyone can get a “good” girlfriend is when women are forced to behave*; banning abortion—*If you weren’t a betacuck, you’d know forcing women to go through pregnancy sends a message to all women to not, “mess around”*). Lack of access to women’s bodies is perceived by Incels as a denial of a “basic right,” something that Chads can effortlessly obtain because “women always give men sex”; it is only Incels that they reject.

As suggested by DeKeseredy and Corsianos (2016), the expectation to engage in high levels of sexual intercourse is common among men, particularly while in college, where peers often encourage these expectations. This idea echoes Elliot Rodger’s motivation and, like in the case of Rodger, when these expectations are not met in offline life, some men feel disappointment, frustration, or anger, and, in some cases, engage in violent behavior (DeKeseredy & Corsianos, 2016). Crucially, these expectations have been linked to the increasing normalization of pornography (Dines, 2010), with its presentation of men with unlimited access to women’s bodies, and women as constantly available sexual objects who never deny men (dehumanizing) sex. Similarly, Incels understand (sexual) relationships as something they deserve and need to gain from women, while these remain passive participants who owe all men (including Incels) sex. Such sense of entitlement to women’s bodies is not limited to Incels or pornography, as shown by the recent ruling of an English judge who sanctioned a man’s “fundamental human right” to “have sex” with his wife (when she no longer has the capacity to consent; Bowcott, 2019). This ruling essentially denied women’s rights to say “no” and refuse unwanted sex, while liberating men from any responsibility.

As shown in the following post, Incels’ perception of themselves as victims, or the only group that is not having sex, springs from this very sense of entitlement to women’s bodies and is used to explain and legitimize their hatred: *Incels have every right to hate women. If not a single one ever showed interest in you [. . .] it means that they dislike you and you have every right to hate people who dislike you*. Clearly, it is their sense of inability to control women’s freedom that leads to their hatred: *I hate ugly women so fucking much. They repulse me and yet they can fuck men way out of there league while they would say no to me*. It is particularly feminists who are blamed. As one user put it: *My inceldom stems from the fact that women have been brainwashed by society, the feminazis. [. . .] Such is the fate of any woman that isn’t subjugated and told what to do.*

Incels often differentiate between the women that they hate and the ones that they do not hate. For instance, one of the most common collocates of *hate* and *women* is *all*. Incels seem to stress the fact that they do not hate *all women*, just some of them. The women they hate are those who refuse to accept their world view, women who say “no,” women who express their sexual freedom in a way that is perceived as disadvantageous for Incels but advantageous for other men and for women themselves, or in Manne’s (2018) words “women who are held to be failing to live up to patriarchal standards” (p. 33). Essentially, Incels hate “feminists”: *I dont hate indian women as a whole. Only those disrespectful stuck up bitches; I don’t hate all women but modern Western women; I don’t hate women just hate feminists and sluts*. Clearly, for Incels, feminism is predominantly linked to sexual freedom. For them, feminism is the reason why women are free to choose the “quantity and quality” of their sexual partners, as well as the reason why their desire to control women—like other men do—is constantly frustrated; therefore, Incels want a return to “traditional” values (e.g., monogamy, no pre-marital sex) as a way to exert the control over women they feel entitled to in view of women’s (presumably natural) subordination to all men, Incels included (Nagle, 2017). The specific hatred for feminists is also shown by the fact that *feminist* is more often associated with (sexually) derogatory language than *woman.* In fact, based on SketchEngine Thesaurus tool, which shows terms used in similar ways in a corpus, *feminist* is similar to words such as *whore, bitch, cuck, slut, idiot, cunt*, while *woman* is more similar to words like *people, man, girl, guy, female, person*. The type of abusive language that one may expect to find on the forum does not target women in general; it tends to be specifically directed toward feminists, who receive insults for their “promiscuity.”

However, it is not women’s “promiscuity” that Incels despise the most, as, for example, they advocate for the legalization of prostitution. What Incels really hate—and what they blame feminists for—is women who refuse *them*, women who sleep with several men but say “no” to Incels. It is these women who receive most online (sexualized) abuse (Lewis et al., 2017), arguably in an attempt to control them through silencing. This generates a paradoxical situation, in which derogatory terms that refer to “promiscuous” women are not being used for women who participate in sexual acts with numerous men, but for women who say “no.”

This is not an uncommon pattern outside the Incels’ world. For instance, research on online reviews of prostitutes (Pettinger, 2011) has shown that men who buy women for sex do not tend to be linguistically abusive toward those who satisfy their requests without objections. In contrast, the women who are given negative reviews are those who refuse to perform acts requested by punters, or those who set boundaries in place for themselves; in other words, women who say “no.” While in prostitution men’s ways of punishing women for not complying with their requests are negative reviews (Pettinger, 2011), online Incels react with overt hatred and abuse. For both, women must be available to satisfy men’s sexual pleasure and those who do not comply must be punished. The ideology that underpins mainstream pornography is not dissimilar either. In pornography, women are also reduced to bodies to be accessed and consumed, feminine performances, rather than people and, while in pornography women’s refusals are made invisible because of the illusion of fiction, all these practices share the aim to silence women and put them “in their place,” one where they always say “yes.” Clearly, these practices share a misogynistic ideology which sees women as *human givers* (Manne, 2018).

### Chads

The analysis of WOMAN has shown that one of the main concerns of Incels is their ability to access women’s bodies. *Who* has sexual access to these is also an issue for Incels. This section presents the analysis of CHAD, the men who are perceived as having effortless access to women’s bodies.

The most frequent nouns modified by CHAD (*cock, thundercock*) show that this category of men is overwhelmingly sexualized and associated with sexual body parts, specifically the penis. The concordance analysis of CHAD and its collocates *cock* and *Thundercock* showed the traits that Incels ascribe to Chads. They are presented as static body parts simply waiting to have sex (e.g., *Who gives a fuck, just jump on the next Chad cock and get bred by it*), and always available (e.g., *you are a female you can get Chad cock 24/7 on demand*). As stereotypical men, Chads are in constant need of sex and, with their perennial sex-craving, devaluation of emotion (e.g., *[Chads] don’t care about your pathetic love poetry or sensitive side*), anti-feminine attitudes which do not stop them from finding (sexually) available women (e.g., *I see chads use, abuse and be very nasty to women and they are still well liked*), dominance and aggression (e.g., *Women desire alpha dominant chad cock!*), Chads epitomize the archetype of hyper-masculinity (Burk et al., 2004), the dominant males Incels aspire to be, but believe they will never be. Descriptions of Chads’ aggressiveness are especially noticeable when Incels discuss Chad’s sexual relationships with women. These are often constructed in aggressive terms, with verbs such as *choking, ravaging, beating, shoving* or *pounding* (*Get the fuck out you fucking roastie, don’t you have Chad cock to get pounded by?; How much you fellas wanna bet the whole time you were taking it in the vag, you were imaging chads giant cock ravaging your insides?; Choke on chad’s dick and die; I’ve never spewed any bullshit in person about any girl wanting 10 inch chad cocks shoved in her face; When chad does wife beating, its just [. . .] sexual foreplay*) or descriptions of aggressive sexual practices such as anal sex (e.g., *Females can’t do anything good except for taking Chad’s cock up their arse*). Here women are again presented as never satisfied or hurt, and in constant need for *Chad’s cock* (e.g., *Once a girl is done riding one Chad, she immediately hops onto the next Chad’s cock*). The latter example, in particular, reiterates the metaphor of women as animals (*hopping* is a verb normally attributed to rabbits or frogs—https://bncweb.lancs.ac.uk).

Often, this type of sexualized language is not just descriptive; it is also an indicator of the presence of abuse and antagonism on the site. In fact, verbal abuse on r/incels is perpetrated through images of dominant and aggressive male sexuality, echoing the weaponization of the penis, and reflecting what Bridges et al. (2010) have defined as some of the most aggressive sexual practices in gonzo pornography, especially anal sex and strangulation. “*Choking*” [sic] or, more precisely, strangulation, in particular, is a form of male violence toward women whose popularity has crossed the borders of mainstream pornography, bleeding into “real life” as a widespread and normalized sexual practice, often referred to as an “erotic game” or “breath play.” In the United Kingdom, this practice is linked to the deaths of a number of women who were killed in what the media (and the law) called “rough sex gone wrong” (Bindel, 2019; Moore & Khan, 2019).

While Incels appear to despise Chads for having sex with as many women as they want to, they also look up to them for their perceived superiority and, indirectly, use their supposed power as a weapon to abuse women. Clearly, (alpha) male sexuality is seen as punishing, revengeful, and violent, but also superior and, therefore, desirable for both women and Incels (e.g., *They are all spoiled to superior chad cock*). The dominance of Chads—embodied by their “success” with women—is presented as an accepted fact and inevitable reality and, as such, less enraging than women’s (“deviant”) “promiscuity.” While Incels feel they could tolerate Chads’ “natural superiority” in terms of “sexual conquest,” they cannot tolerate this in women because of their “naturally inferior” status, not only to Chads (Nagle, 2017) but also to Incels (in that they are men too). In other words, to reduce the impact of Chads’ dominance on Incels’ lives, it is women, not Chads, who—due to their “inferiority”—can and should be restrained. While Incels feel they have to compete with other men to gain sexual access to women, they also feel that this fight is not about controlling other men, but about controlling women. However, this sense of entitlement is constantly frustrated, as Incels feel unable to possess women like alpha men do, due to women’s refusal to do the emotional labor expected of them (e.g., *They all have the potential to cure you of your virginity, but they refuse to instead opting to take chad’s cock*) by denying (emotionally available) Incels what they give to (emotionally unavailable) alpha men (e.g., *You were a fucking slut that wanted chad cock but did not get it so you refused all the other incels that wanted to be romantic with you*). Incels seem to hate women because they remind them of their own “weakness” compared to other men. At the same time, due to the acceptance of Chads’ “superiority” and awareness of Incels’ “inferiority,” and in line with men’s general reluctance “to tell other men what they can and cannot have sexual access to” (MacKinnon, 1984, p. 333), Incels do not direct their disgust toward Chads, but toward women.

In fact, it is women’s “promiscuity” that is condemned, not Chads’ (e.g., *Plus, 95% of girlfriends have had 30+ chad cocks prior to being with that boyfriend, so she is used goods anyway*), and what seems to be particularly abhorrent for Incels is the idea of having a female sexual partner who has already had (numerous) sexual partners (especially Chads). For example, as shown in the following example, they use the word *cuck*—a man who is “weak” and has “failed” because he has accepted to start a (sexual) relationship with a woman despite his awareness that she had sexual intercourse with a Chad—as potentially the worst abuse for men: *Anytime you start liking a female, imagine her sucking the cum out Chad’s cock. [. . .] Imagine all the Chad’s cocks that have been in her hole. [. . .] imagine having to touch all the AIDS infested Chad cum residues on her. If you’re into that shit, you are a cuck and should kill yourself*. Incidentally, *cuck* also refers to any man whose woman is having sex with another (alpha) male, and, as very often this alpha male is Black, the word has a clear racialized dimension too. This theme is very common in pornography, where “cuckhold porn”—which typically depicts a White man “forced” to watch his White wife have sex with a Black man—has become a popular subgenre and has provided the “framing narrative” for groups in the manosphere to “express masculinist and racist ideologies” (Lokke, 2019, p. 213). It is worth noting, though, that on the Incel “alpha scale” (and, arguably, in mainstream pornography too), White men would still be classed as superior. The Black alpha male is so only because he is reduced to stereotypes about his appendage, but the racial inequalities apply within the Incel community (and mainstream pornography) as they do in wider society.

The idea of women being “used goods” permanently “marked” by Chads’ semen parallels a pornographic practice in which semen is held in women’s orifices and shown to the audience before dribbling down their bodies (Dines, 2010). Incels’ revulsion for these women, together with their insecurity for lacking sexual experience, further motivates Incels’ support for traditional notions of women’s virginity and chastity (e.g., *I am a virgin, and so I am not interested in a woman that took 50 Chad cocks inside her already*) and reproach of feminism for causing a loss of women’s “purity” (e.g., *Yeah, blowing 3 Chad cocks and jerking off 2 under an hour is very empowering*). Incels’ aversion for women’s sexual freedom is also evidenced in their in-group slang, for example, with the word *roastie*, which, according to Urban Dictionary, describes sexually “promiscuous” women, in reference to their labia resembling “roast beef” because of frequent sexual intercourse (“Roastie,” n.d.).

The men that Incels perceive as being Chads and the women that have sex with them resemble, in many ways, the men and women in pornography. For example, Incels’ linguistic abuse of women through sexual degradation betrays hyperbolic views about their sexuality that are also common in gonzo pornography, like the obsession with women’s “promiscuity” or the idea that women “beg for sex” (e.g., *Soon the roastie will run out of Chad cock, and her deranged mind will suffer; They will only mature into cumsluts who will beg for Chad’s cock daily and will continue to make Incel’s lives hell*). Similarly, Chads resemble the hypersexualized men of mainstream pornography, particularly Black men who, according to Dines (2010) “are fast becoming [. . .] the most sought after ‘fuckers’ of white women” (p. 285). Incels often discuss Black men as the reason for their “inceldom,” while White women who only date Black men—and reject Incels—are linguistically abused with distinctive terminology: *mudshark* (e.g., *Meanwhile almost every white single mother I see is a mudshark, atleast 90%, so I know that if it wasn’t for black men I would be far less likely to be an incel*). Like *roastie* and *cuck*, these specific terms suggest the standardization of these concepts for Incels.

### Fuck

The language used by Incels to talk about sexual intercourse (particularly between Chads and women) mirrors the aggressiveness of sex in mainstream pornography, as further shown by the discourse surrounding the verb *fuck* on r/incels. The only collocate of *fuck*, excluding swearing, with an overall score higher than 10 is the pronominal object *her*, particularly in the expression *to fuck her*.

The discourse surrounding *fuck* further elucidates how Incels conceptualize sex as a form of punishment for women, not simply as imposition, but as an actual form of physical harm and degradation. This is clearly articulated in the following comments:

*You don’t even know how hard you could fuck her; that’s why you fuck her again and don’t stop until she miscarries; Thoart fuck her. I want her make up running down her face. Ill probably cum in her a few times; I think she was into you. You should have whipped out your dick and fucked her; You can just fuck her and degrade and mistreat her so she doesn’t get under the impression you respect her in any way; I’d definitely fuck her in the ass and then make her suck my shit-covered dick.*

Like in offline sexualized violence, for Incels too sex is the chosen weapon to express the hatred and revenge that women “deserve” for rejecting them and choosing aggressive alpha men instead. These scenarios are presented in the conditional tense, as hypothetical situations that are about what Incels *would, should, or could do*, rather than what they *did* or *are doing*. In some cases, the tense is imperative (*throat fuck her*), suggesting a male bond created through encouragement and peer support for other men to be aggressive with women (DeKeseredy & Corsianos, 2016).

These examples confirm Incels’ desire to replicate or imitate what is seen as Chads’ alpha male punitive behavior with women, as well as their sense that Incels will never be able to do the same. Yet, like above, what angers Incels the most is women’s freedom and rejection of Incels. As one user put it: The temerity of modern western WHORES is beyond all reason. It infuriates me just THINKING about a woman cruelly REJECTING me with her body posture and attitude, as if saying “Don’t even come near me” while she still has **CHAD’S** cum dripping out her fucked out anus. Punishing women for choosing Chads over Incels is the priority; they would rather see them “punished” by Chads than free. To this end, the types of hypermasculine behavior that Incels could and would adopt if they were Chads include sexualized violence (e.g., *Grab a girl straight from the street drag her into a restroom and fuck her, it will be consensual sex, since I am chad*). Rape apology appears on r/incels as part of a broader discussion of sex as power, as sex is the weapon used to inflict the abuse that women both “deserve” and “enjoy.” They describe scenes of slapping, anal sex, ejaculating on women’s faces, and multiple and aggressive penetration of different orifices, including from anus to mouth without washing.

Here, these images can appear to be “extreme,” but elsewhere they are normalized. For example, despite their direct origin in misogynistic pornography, they are predominant in the definitions provided by Urban Dictionary, the 22^nd^ most popular site in the United States and one of the most prominent results of Google searches for word definitions (Ging et al., 2019). Furthermore, in mainstream pornography, a multibillion industry with over four million sites on the internet (DeKeseredy & Corsianos, 2016), these images are glorified as adult entertainment and erotic practice, and they are considered neither “extreme” nor “fringe.” They are the norm. In fact, vaginal, anal, and oral penetration of a woman by multiple men at the same time, gagging—in which a woman has a penis thrust so far down her throat she gags (or vomits)—ATM, “bukkake,” “money shot,” are among the most popular scenes in gonzo pornography (Dines, 2010, p. xxvii). Thus, while the harm of Incels is made very visible in name of their “extreme” beliefs and actions (although these are mostly seen as the result of their individual, pathological deviations, rather than linked to structural misogyny; Manne, 2018), the harm of pornography has been rendered invisible, as its content and consumption become increasingly normalized (yet not normal). But invisible does not mean nonexistent. Pornography is not harmless; its harm exists and resides, partly, in its insidious ability to infiltrate (while being infiltrated by) mainstream culture. This article has sought to make its misogynistic nature visible by focusing on its similarities with discourses which are socially recognized as harmful.

## Conclusion

In both Incel and mainstream pornography discourse, much of the denigration of women focuses on their sexuality. Their imagery and language present women as objects who deserve and enjoy sexual abuse and submission, and sex (particularly through the penis and semen) as a weapon to inflict these and express their hate. On r/incels the fact that hatred is the motivation behind the abuse is explicit. In pornography, this motive is often covert and, consequently, easier to justify. However, it sometimes filters through. For example, according to Bill Margold—porn actor and producer—“the most violent we can get is the cum shot in the face. Men get off behind that, because they get even with the women they can’t have” (Dines, 2010, p. xxvi). These words resonate with Incels’ desire to cause women pain for preferring Chads to them and use sex to get even with women that they feel they will never have. Furthermore, the imagery of abuse and discourses of masculinity used by some popular porn sites to advertise themselves disturbingly resemble the violent descriptions of sexual intercourse with women and the traits ascribed to Chads by Incels: “Do you know what we say to things like romance and foreplay? We say fuck off! [. . .] We take gorgeous young bitches and do what every man would REALLY like to do. We make them gag till their makeup starts running, and then they get all other holes sore” (Dines, 2010, p. xix). In this sense, we argued, the men in pornography are the embodiment of Chads. They have constant access to women despite hating them and treating them badly. While Incels despise and envy Chads for this, for them these men (and their dominant sexuality) are the only way to obtain their revenge. What all these men have in common is the wish to see women suffer through sex, while drawing pleasure and satisfaction from it.

Similarly, men who watch pornography, as shown by Dines (2010, p. 89), “feel like *sexual losers*. They thought *college* would present easy opportunities for sex, assume that *other guys are ‘getting it’*, and conclude that something must be amiss with them or the women they are trying to hook up with. They worry they are *not good-looking enough*, smooth enough, or masculine enough to score, and as the porn view of the world suggests that *women are constantly available*, these men are bewildered by rejection. They often express deep *shame* about their inability to hook up, and this shame morphs into *anger at their female peers* who, unlike porn women, have the word ‘no’ in their vocabulary” (emphasis added). Dines’s words describing these men worryingly echo the justification that Elliot Rodger gave for his killing spree and the discursive patterns found in the analysis of r/incels.

Studying the discourse of r/incels allowed us to unveil the ideological context that underpins this community, to identify how assumptions that underpin “extreme” discourse are also present within the mainstream majority, and to demonstrate how these flow into each other in a mutual process of production and reproduction of language, behaviors, and attitudes. While perhaps less pervasive and persistent than other forms of misogyny, Incels’ sexism is one of the faces of societal misogyny based on biological determinism and a binary gender distinction. It is rooted in the same misogyny of ordinary sexist jokes, assumptions, everyday division of labor, and media representations, including mainstream pornography and, for this reason, should not be conceptualized as exceptional or unusual. By continuing to focus on what makes the community “extreme,” the risk is ignoring what makes it similar to the mainstream, thus contributing to the belief, often spread by mainstream media, that misogyny is only a problem of specific individuals and their specific circumstances (Tranchese, 2019, 2020), rather than also linked to institutionalized misogyny, ingrained in the fibers of society. In fact, while some members of the Incel community have committed acts of VAW, informed by their misogynistic attitudes, most crimes against women are not performed by members of this community, but by the mainstream majority. As suggested by Connell (2005), “[o]n a world scale, explicit backlash movements are of limited importance, but very large numbers of men are nevertheless engaged in preserving gender inequality” (pp. 1816–1817).

The issue, therefore, is a broader one and a cultural one; neither Incel ideology nor mainstream pornography should be problematized separately or insulated from discussions of women’s equality, because these practices are not detached from other forms of misogyny but are an extension of these and symptoms of structural misogyny. Neither pornography nor Incels created misogyny, but misogyny underlies and correlates both practices, which, therefore, should be understood as part of a “networked misogyny” (Banet-Weiser & Miltner, 2016) that, separately or cumulatively, causes harm and is not limited to the online world. While the internet, as a “site of social and cultural reproduction that reflects real-world patterns” (Lewis et al., 2017, p. 1464), enables the exponential replication of misogyny by inventing, spreading, and reproducing techniques to attack women (online and offline), online misogyny is not a product of the technology, but a result of the society that shaped it.

Taken separately, pornography presents itself as fiction and, therefore, harmless, but highlighting its similarities with misogynistic hate speech challenges its legitimation. It is therefore necessary to question whether labels that create and reinforce this division (like “extreme” or “dangerous”) are appropriate for and can justifiably be applied to more reproached practices if they are not also ascribed to other practices that, while normalized because routine, still draw on the same ideological context. In fact, pornography, like Incels’ misogyny, can hurt individuals, “just not as individuals in a one-at-a-time sense, but as members of the group ‘women’” (MacKinnon, 1984, p. 338). Part of the harm caused by these practices is the way in which their language spreads across genres and permeates society. It is not necessary to actively watch pornography to be able to use this language (some men on r/incels deny watching it), because, as shown by the analysis of r/incels, mainstream pornography legitimizes and propagates a discourse which, while rooted in societal misogyny, spreads online and offline (thanks to the virtually unlimited accessibility of online pornography), and upon which Incels can draw to (sexually) degrade women (Dines, 2010). At the same time, while Incels are provided with the language and imagery, or a “script,” to express their hatred, they can also transmit their own “creative” ways of expressing hatred against women beyond their group, both online and offline (Jane, 2016). However, the harm caused by both practices often varies in terms of visibility. Incels’ harm is often flamboyant, while pornography’s harm is often unperceivable; yet, it is precisely because of its invisibility that this harm is so pervasive.

Whether more or less visible, the damaging effects of both pornography and Incels’ misogyny can be lasting because of the longevity and visibility that the internet grants violent narratives and abusive texts (Lewis et al., 2017). Traces of abuse remain and can be read by thousands of other men. But their perlocutionary effect does not end with men building a group identity through attacks directed toward women; it continues as a warning for women that, ultimately, aims to silence them and keep them in “their place.” Like in offline VAW, in the manosphere, of which mainstream pornography can be considered a part given its parallels with Incels’ discourse, men also express a “territorial exclusivity” (Lewis et al., 2017, p. 1464) to determine the boundaries of what women can or cannot do, or the (online or offline) spaces that women can or cannot inhabit. Women are only accepted in these spaces if they bring men pleasure, like in pornography, where women stay “in their place” to fulfill men’s desires, and always say “yes,” particularly to degrading, violent, and dehumanizing practices. Yet, when women try move out of “their place” to (metaphorically) reclaim their own space and establish their own (sexual) boundaries, when they refuse to abide, they are punished in various ways (physically, sexually, with negative reviews, threats, verbal abuse, exclusion). In fact, it is particularly dissenting women, “visible and audible” (Lewis et al., 2017, p. 1462) women who dare to leave the position of subordination in which patriarchal societies have historically relegated them, women who refuse to be extensions of men but people in their own right, or, fundamentally, women who are perceived as being feminists, that attract high levels of violence and eminently feature at the receiving end of online and offline abuse (Lewis et al., 2017, p. 1465). As conveyed by pornographic actor, producer and director Max Hardcore—echoing, inspiring, or in unison with Incels—“I don’t hate all women, just stuck up bitches” (Dines et al., 2013, p. 81). While the exclusion of these women is represented and enacted in pornography, as women do not find space in it except to be degraded, in the manosphere—and in society more broadly—violence or exclusion are fears women are threatened with, inculcated into them as people without authority in a world that is male territory.
